# 25(OH)VitD and human endocrine and functional fertility parameters in women undergoing IVF/ICSI

**DOI:** 10.3389/fendo.2022.986848

**Published:** 2022-08-29

**Authors:** Mei Tian, Suimin Zeng, Sufen Cai, Christoph Reichetzeder, Xiaoli Zhang, Chenjun Yin, Weihong Kuang, Kexin Cheng, Yao Jiang, Mingqiu Tao, Yuan Zeng, Ge Lin, Jian Li, Fei Gong, Berthold Hocher

**Affiliations:** ^1^ Key Laboratory of Study and Discovery of Small Targeted Molecules of Hunan Province, School of Medicine, Hunan Normal University, Changsha, China; ^2^ Department of Pathology, The First Hospital of Traditional Chinese Medicine, Yiyang, China; ^3^ Clinical Research Center for Reproduction and Genetics in Hunan Province, Reproductive and Genetic Hospital of CITIC-Xiangya, Changsha, Hunan, China; ^4^ Institute of Reproductive and Stem Cell Engineering, School of Basic Medical Science, Central South University, Changsha, China; ^5^ Department of Nutritional Toxicology, Institute for Nutritional Science, University of Potsdam, Nuthetal, Germany; ^6^ Institute of Pharmacy, Freie Universität Berlin, Berlin, Germany; ^7^ Fifth Department of Medicine (Nephrology/Endocrinology/Rheumatology), University Medical Centre Mannheim, University of Heidelberg, Mannheim, Germany; ^8^ Key Laboratory of Stem Cells and Reproductive Engineering, Ministry of Health, Changsha, China; ^9^ Institute of Medical Diagnostics, IMD, Berlin, Germany

**Keywords:** free 25-hydroxyvitamin D, total 25-hydroxyvitamin D, *in vitro* fertilization and embryo transfer, fertility, endocrine

## Abstract

**Background:**

Vitamin D plays an important role in reproduction. Evidence shown that free 25-hydroxyvitamin D (25(OH)VitD) was more accurate than total 25(OH)VitD in reflecting the status of 25(OH)VitD during pregnancy. However, the relationship between free 25(OH)VitD and female fertility parameters has not been reported yet. Therefore, this study aims to compare the correlation of free and total 25(OH)VitD with fertility parameters in infertility females undergoing *in vitro* fertilization and embryo transfer (IVF-ET) or intracytoplasmic sperm injection (ICSI).

**Methods:**

According to the inclusion and exclusion criteria, 2569 infertility patients who received IVF-ET or ICSI treatment for the first time participated in this study. Five milliliter peripheral blood samples of the patients were collected on the day before embryo transfer (ET). Enzyme linked immunosorbent assay (ELISA) kits was used to detect free 25(OH)VitD and total 25(OH)VitD, and clinical information was collected. Spearman’s rho was used to evaluate the association between the variables.

**Results:**

The median (IQR) of free 25(OH)VitD was 4.71 (4.11-5.31) pg/mL and total 25(OH)VitD was 19.54 (16.52-22.83) ng/m. The correlation between them, however, was week (rho=0.311). Compared to total 25(OH)VitD, free 25(OH)VitD was slightly better correlated with basal follicle-stimulating hormone (FSH) (rho=0.041, *P*=0.036), basal estradiol (E_2_) (rho=0.089, *P*<0.001), anti-Müllerian hormone (AMH) (rho=-0.057, *P*=0.004), antral follicle count (AFC) (rho=-0.053, *P*=0.007), E_2_ (rho=-0.080, *P*<0.001), number of oocytes retrieval (rho=-0.079, *P*<0.001) and progesterone (P)/E_2_ on hCG trigger day (rho=0.081, *P*<0.001).

**Conclusions:**

Overall, there was only a rather weak correlation of free as well as total 25(OH)VitD with human endocrine and functional fertility parameters in women undergoing IVF/ICSI. Neither free nor total 25(OH)VitD seems to play a major role in human embryo implantation.

## Introduction

Infertility affects more than 123 million women worldwide (1), and about 15% women of childbearing age in China suffer from infertility (2). More and more infertility patients have their own offspring through assisted reproductive technology (ART) (3). *In vitro* fertilization-embryo transfer (IVF-ET) is one of the most effective techniques for treating infertility. Controlled ovarian hyperstimulation (COH) is using ovulation drugs to induce the development and maturation of multiple follicles within a controllable range, which is usually used to obtain more high-quality mature oocytes in IVF-ET (4). Ovarian response to COH can be divided into normal response and abnormal response, which are related to ovarian reserve function. During the ART treatment, evaluating the fertility of patient, including ovarian reserve, uterine condition and systemic factors, is of great significance to improve pregnancy success rate (5).

Vitamin D is a fat-soluble steroid derivative, which is bound to vitamin D-binding protein (DBP) in circulation, then form 25-hydroxy vitamin D (25(OH)VitD) in the liver and turn to 1,25-dihydroxyvitamin D (1,25(OH)_2_D) in the kidney (6). The majority of vitamin D and its metabolites are tightly bound to DBP, and about 10%~15% of these metabolites bound to albumin, while less than 1% of circulating vitamin D metabolites circulate in a free, unbound form. Free 25(OH)VitD can pass the lipophilic cell membranes and interact with the nuclear vitamin D receptor. Albumin-bound vitamin D is relatively easily available after dissociation from albumin because it is bound less tightly to 25(OH)VitD as compared to the binding of 25(OH)VitD to DBP. The sum of free and albumin-bound forms is called bioavailable vitamin D. Total 25(OH)VitD is defined as the sum of free, albumin-bound and DBP-bound 25(OH)VitD, respectively (7).

In addition to its classic role in regulating calcium and phosphorus levels in bone metabolism, it is closely related to female reproduction and participate in the process of follicle development, sex hormone production and embryo implanting (8–11). Studies have confirmed that human endometrium, ovarian and placenta and other female reproductive organs have vitamin D receptors (VDR) (12). Animal experiments showed that when VDR gene was knocked out, female mice showed thinner endometrium and smaller ovaries, followed by dysplasia of follicles and no luteinization, indicating that vitamin D plays an important role in maintaining the normal development of the uterus and ovaries (13). Multiple studies have shown that infertility patients have vitamin D deficiency (serum total 25(OH)VitD < 20 ng/mL), and further studies have found that 25(OH)VitD levels have a significant impact on the fertility, which suggest that 25(OH)VitD may affect the fertility of patients with infertility (14–17).

At present, serum total 25(OH)VitD is a general indicator used to measure the 25(OH)VitD status in clinical practice, but free 25(OH)VitD, rather than total 25(OH)VitD, can enter cells to produce biological activity. A recent study by our team found that free 25(OH)VitD is more accurate than total 25(OH)VitD in reflecting the status of 25(OH)VitD during normal pregnancy (18). Although there are many studies reporting the effects of 25(OH)VitD on reproduction, while very few studies on the role of free 25(OH)VitD in reproduction. Recent research have suggested that measuring free 25(OH)VitD instead of total 25(OH)VitD might be a superior measure of 25(OH)VitD status (18–20).

In a recent huge prospective clinical observational study, we have measured free and total 25(OH)VitD concentration in women undergoing fresh embryo transfer and analyzed its association with early pregnancy outcome parameters such as biochemical pregnancy, implantation rate, and clinical pregnancy rates, while the results showed that neither free nor total 25(OH)VitD was associated with successful embryo implantation (21). To get a better understanding of this unexpected result, we now did a *post-hoc* analysis of our data analyzing the relationship between 25(OH)VitD level and female endocrine and functional fertility parameters in our large southern Chinese ART cohort.

## Materials and Methods

### Study design and setting

This was a *post-hoc* analysis of a single-center observational study (21–23). We had invited infertile women undergoing their first IVF cycles or intracytoplasmic sperm injection (ICSI) from January 2017 to December 2018 at the Reproductive and Genetic Hospital of CITIC-Xiangya in Changsha, Hunan Province, China. Our study was approved by the Ethics Committee of the Reproductive and Genetic Hospital of CITIC-Xiangya (approval number: LL-SC-2018-014), and informed written consent was obtained from every participant. All the patients considered for ART in our center underwent B-ultrasound screening. The clinical data of patients were obtained from the hospital’s electronic patient`s records.

### Study population

Women undergoing first IVF/ICSI cycles were invited to participate in this study. A total of 2569 infertile patients’ information were extracted from the electronic medical record in the hospital. The inclusion criteria were (1): age between 20 and 38 years old (2), received fresh embryo transferred. Exclusion criteria were (1): endometriosis (2), uterine malformations (3), uterine myoma (multiple, submucous or intramural myoma >3 cm) (4), untreated hydrosalpinx (6), oocyte donation cycles (7), pre-implantation genetic test (8) Cushing syndrome (9), adult-onset adrenogenital syndrome (10), any hypothalamic or pituitary disease leading to infertility (21).

### Clinical data collection of the participants

(1) The general information of the patients were collected, including age, ethnicity, education level, years of infertility, type of infertility, body mass index (BMI), random blood glucose, pulse and blood pressure.
(2) Baseline data: basic follicle-stimulating hormone (FSH), basic luteinizing hormone (LH), basic FSH/LH, basic estradiol (E_2_), basic progesterone (P), basic P/E_2_, basic prolactin (PRL), basic testosterone (T), anti-Müllerian hormone (AMH), antral follicle count (AFC) and basal endometrial thickness.
(3) COH outcome: COH regimen, gonadotropin (Gn) dosage, human chorionic gonadotropin (hCG) dosage, LH, E_2_, P, PRL and endometrial thickness on hCG trigger day.
(4) The indicators of the day before ET: E_2_, P, PRL and endometrial thickness on the day before ET.

### Measurements and sample collection for total and free 25(OH)VitD

Five milliliter peripheral blood samples of the patients who got at least one embryo suitable for embryo transfer (ET) were collected on the day before ET. After centrifugation, the serum was taken and stored at -80°C. Total 25(OH)VitD was detected by means of Enzyme linked immunosorbent assay (ELISA) with ELISA kit (DIAsource ImmunoAssays S.A., Belgium) according to the instructions. Free 25(OH)VitD concentrations were measured with ELISA kit (Future Diagnostics Solutions B.V., Netherlands) (18, 24).

According to international guidelines, concentration of total 25(OH)VitD <20 ng/mL was categorized as deficiency, between 20–30 ng/mL was categorized as insufficiency, while concentration of total 25(OH)VitD >30 ng/mL was categorized as adequate (25, 26).

### Data analysis

Statistical analysis was performed using Statistical Package for Social Science (SPSS) for Windows (Version 20.0 software, SPSS Inc., Chicago, IL, USA). Values were presented as medians (interquartile ranges) or frequency (n %). A comparison of quantitative variables among groups about female fertility parameters was performed with the Kruskal-Wallis test. The P-value less than 0.05 was considered significant.

## Results

According to the inclusion and exclusion criteria, 2569 women who received IVF-ET treatment for the first time participated in this study. The characteristics of the study population are presented in **Table 1**. The key characteristics are typical for a Chinese ART population.

**Table 1:** Parameter of the study population.

Parameter	Values (Medium (IQR) or n (%))
Age (years)	29.00 (27.00-32.00)
Han nationality	2125 (82.72%)
Smoking history	31 (1.21%)
Drinking history	2 (0.08%)
Education levels	
Primary school and below	65 (2.53%)
Junior, secondary and high school	1557 (60.65%)
College and above	945 (36.81%)
Duration of attempt to conceive (years)	3.00 (2.00-5.00)
Previous conception	1203 (46.83%)
BMI (kg/m²)	21.33 (19.65-23.05)
Number of pulse (times/minute)	80.00 (76.00-88.00)
Fasting blood sugar (mmol/L)	5.18 (4.94-5.45)
Blood pressure (mmHg)	
Systolic blood pressure (mmHg)	115.00 (108.00-122.00)
Diastolic blood pressure (mmHg)	76.00 (70.00-81.00)
Basal FSH (mIU/mL)	5.62 (4.79-6.54)
Basal LH (mIU/mL)	3.59 (2.62-4.98)
Basal FSH/LH	1.57 (1.11-2.15)
Basal E_2_ (pg/mL)	33.00 (27.00-43.00)
Basal P (ng/mL)	0.24 (0.17-0.32)
Basal P/E_2_	7.20 (4.81-10.48)
Basal PRL (ng/mL)	14.79 (11.03-19.89)
Basal T (ng/mL)	0.28 (0.22-0.36)
AMH (ng/mL)	5.69 (3.66-9.30)
AFC	24.00 (16.00-30.00)
Basal endometrial thickness (mm)	8.10 (6.40-10.00)
E_2_ on the day before ET(pg/mL)	1008.00 (712.85-1335.00)
P on the day before ET (ng/mL)	60.00 (58.63-60.00)
P/E_2_ on the day before ET	56.66 (43.38-78.47)
PRL on the day before ET (ng/mL)	41.33 (30.88-53.80)
Endometrial thickness on the day before ET (mm)	13.30 (12.00-14.70)

BMI, body-mass index; FSH, follicle-stimulating hormone; LH, luteinizing hormone; E_2_, estradiol; P, progesterone; PRL, prolactin; T, testosterone; AMH, anti-Müllerian hormone; AFC, antral follicle count. ET, embryo transfer; E_2_, estradiol; P, progesterone.

Free and total 25(OH)VitD on the day before ET was not normally distributed (*P*<0.05). The median (IQR) of free 25(OH)VitD was 4.71 (4.11-5.31) pg/mL and total 25(OH)VitD was 19.54 (16.52-22.83) ng/mL. The correlation between free 25(OH)VitD and total 25(OH)VitD was week (rho=0.311). According to the difference in the concentration of free 25(OH)VitD and total 25(OH)VitD, patients were divided into three groups. There were statistical differences in basic LH, E_2_ and E_2_, P/E_2_, the number of eggs obtained on the hCG trigger day among the three groups of free 25(OH)VitD (*P*<0.05). Paired comparison results showed that the basic E_2_ of the F3 group (free 25(OH)VitD≥ 5.11 pg/mL) was significantly higher than that of the F1 (free 25(OH)VitD< 4.32 pg/mL) and F2 groups (4.32 pg/mL < free 25(OH)VitD< 5.11 pg/mL) (*P*<0.05), while E_2_ on the hCG trigger day in the F3 group was significantly lower than that of the F1 group, and the P/E_2_ and the number of eggs obtained on the same day were significantly higher (*P*<0.05). (**Supplementary Table S1**)

There were statistically significant differences in the amount of hCG, LH, E_2_ and endometrial thickness on the day of hCG trigger day among the three groups of total 25(OH)VitD (*P*<0.05). Compared with the T1 group (total 25(OH)VitD< 20 ng/mL), the T2 group (20 pg/mL < total 25(OH)VitD< 30 ng/mL) had significantly higher basic E_2_, LH, E_2_ on the hCG trigger day and lower endometrial thickness on the hCG trigger day (*P*<0.05). And the T3 group (total 25(OH)VitD≥ 30 ng/mL) had significantly lower hCG dosage and higher E_2_ on the hCG trigger day than that of the T1 group (*P*<0.05) (**Supplementary Table S1**).

Spearman analysis showed that free 25(OH)VitD was positively correlated with the basal FSH (rho=0.041, *P*=0.036), E_2_ (rho=0.089, *P*<0.001) and P/E_2_ on the hCG trigger day (rho=0.081, *P*<0.001), and was negatively with AMH (rho=-0.057, *P*=0.004), AFC (rho=-0.053, *P*=0.007), E_2_ on hCG trigger day (rho=-0.080, *P*<0.001) and numbers of oocytes retrieval (rho=-0.079, *P*<0.001) (**Table 2**). The total 25(OH)VitD was positively correlated with age (rho=0.060, *P*<0.01), basal E_2_ (rho=0.068, *P*<0.01), LH (rho=0.070, *P*<0.001) and E_2_ on the hCG trigger day (rho=0.072, *P*<0.001), and was negatively correlated with basal FSH/LH (rho=-0.039, *P*<0.05) (**Table 2**).

**Table 2:** Analysis and comparison of the correlation between free and total 25(OH)VitD with fertility parameters and COH outcomes in IVF-ET females.

Parameter, units	Free 25(OH)VitD (pg/mL)	Total 25(OH)VitD (ng/mL)	Spearman's rho color gradient
E_2_ on hCG trigger day (pg/mL)	-0.080^***^	0.072^***^	-0.05>rho≥-0.08, p<0.05
Number of oocytes retrieved	-0.079^***^	-0.023	-0.03>rho≥-0.05, p<0.05
AMH (ng/mL)	-0.057^**^	0.025	0.00>rho≥-0.03, p>0.05
AFC	-0.053^**^	0.017	0.00≤rho<0.04, p>0.05
Basal P/E_2_	-0.033	-0.008	0.04≤rho<0.09, p<0.05
Basal FSH/LH	-0.020	-0.039^*^	
Basal PRL (ng/mL)	-0.007	0.000	
Endometrial thickness on hCG trigger day (mm)	-0.006	-0.036	
Basal T (ng/mL)	-0.004	-0.010	
PRL on hCG trigger day (ng/mL)	-0.002	-0.009	
Age (years)	0.000	0.060^**^	
Basal endometrial thickness (mm)	0.007	-0.025	
LH on hCG trigger day (pg/mL)	0.011	0.070^***^	
Basal P (ng/mL)	0.018	0.027	
P on hCG trigger day (ng/mL)	0.020	0.011	
Basal LH (mIU/mL)	0.032	0.036	
Basal FSH (mIU/mL)	0.041^*^	0.023	
P/E_2_ on hCG trigger day	0.081^***^	-0.034	
Basal E_2_ (pg/mL)	0.089^***^	0.068^**^	

25(OH)VitD, 25-hydroxy25(OH)VitD; COH, controlled ovarian hyperstimulation; Gn, gonadotropin; hCG, human chorionic gonadotropin; LH, luteinizing hormone; E_2_, estradiol; P, progesterone; PRL, prolactin.

Data are shown as Spearman’s rho value.

^*^P<5, ^**^P<0.01, ^***^P<0.001.

Compared with total 25(OH)VitD, free 25(OH)VitD was more correlated with basal FSH, basal E_2_, AMH, AFC, E_2_ and P/E_2_ on hCG trigger day and numbers of oocytes retrieval (**Table 2**).

## Discussion

Human reproduction is a process that consists of many steps, the ovary produce mature oocytes, which should be picked up by the oviduct where oocyte meet the mature, then the created embryo moves to the uterine cavity and then must be implanted in the endometrium (27). In reproductive medicine interventions, factors that influence the individual steps can be studied separately. In our study, we focused on embryo implantation, as this is the critical step in reproductive medicine (28). Failure of a reproductive procedure is usually due to the unsuccessful implantation of the embryo.

In a recent huge prospective clinical observational study (21), we have measured free and total 25(OH)VitD concentration in women undergoing fresh embryo transfer and analyzed its association with early pregnancy outcome parameters such as biochemical pregnancy, implantation rate, and clinical pregnancy rates, while the results showed that neither free nor total 25(OH)VitD was associated with successful embryo implantation. However, the reason for this remained unclear. Therefore, in this study, we analyzed the influence of 25(OH)VitD on endocrinological and functional parameters of embryo implantation. The association with important hormones of human reproduction as well as functional parameters were detectable, but overall, of marginal importance. These data are consistent with the clinical results of our study.

The results of this study as well as our clinical endpoint study are unexpected in that there are a large number of published studies assign 25(OH)VitD an important role in reproduction (29–34). Our study was certainly not too small. We analyzed over 2500 reproductive medical procedures. The majority of previously published studies on 25(OH)VitD and reproductive medicine that reported an association between vitamin D deficiency and failures in human reproduction were much smaller. However, the only study that analyzed implantation failure fit with our data showing no relevant relationship (35).

Some points need to be addressed. Certainly, there is a publication bias that promotes publications that assume such an association between vitamin D and human fertility. One recent study showed that vitamin D concentration in follicular fluid of infertility patients undergoing ovarian stimulation varies according to the developmental stage of the oocyte and was associated with embryo development status (36). A prospective cohort study included 150 infertile women who underwent IVF or ICSI found that women with higher levels of vitamin D in their serum and follicular fluid are significantly more likely to achieve pregnancy but without affecting the quality of embryo and fertilization rate (37). These studies differ from our results, possibly due to differences in sample size and study design. We focused in our study on the embryo implantation step - the most clinically critical step. Thus, our study only says something about this step of reproduction. Whether e.g. 25(OH)VitD plays a role in oocyte maturation, fertilization, embryo development before implantation, growth of the implanted embryo etc. cannot be deduced from our data. Our studies only show that the role of 25(OH)VitD in implantation seems to be marginal.

Our data are robust, we have determined both free 25(OH)VitD and total 25(OH)VitD in a relatively huge study population, Besides the overall weak correlation of free and total 25(OH)VitD with endocrine and functional parameters of human fertility, there was likewise a weak correlation between free and total 25(OH)VitD. This is most likely due to factors specific to reproductive medicine: ovarian hyperstimulation, which leads to a strong stimulation of estradiol, which itself stimulates the synthesis of sex hormone binding protein (SHBG) in the liver (38). Studies found that 25(OH)VitD and SHBG were significantly associated (39, 40). The response of women to the hyperstimulation of the ovary is highly variable (41, 42), so that ultimately the hepatic synthesis of 25(OH)VitD binding protein is also highly variable and thus explains the relatively poor correlation between free and total 25(OH)VitD, because the free 25(OH)VitD is not dependent of concentrations of SHBG. Our recently published study in young healthy women aiming to have children without ART clearly shows a clinically relevant association between female hormones relevant to reproduction and 25(OH)VitD (43). The key difference here is that these women have not undergone ovarian hyperstimulation. Under the conditions of ovarian hyperstimulation, the importance of 25(OH)VitD in contrast to the woman’s natural menstrual cycle obviously recedes.

The sample size of this study is considerable, but there are still some shortcomings. Firstly, it was a single-center study, and the research was performed mainly in Chinese Han population. Secondly, we did not analyze hormones of the glucose status although vitamin D plays a key role in controlling insulin resistance in women and is disturbed in women with polycystic ovary syndrome (44, 45). Thirdly, vitamin D was not prescribed to the patients in the study, however, we could not control their own intake of vitamin D containing food supplements. Finally, we measured free and total 25(OH)VitD but not 1,25(OH)_2_D, which is produced in the human placenta besides the kidneys and may have different biological effects as compared to 25(OH)VitD (7).

## Conclusion

Overall, there was only a weak correlation of free as well as total 25(OH)VitD with human endocrine, including basal FSH, basal E_2_, AMH, AFC, E_2_ and functional fertility parameters, such as P/E_2_ on hCG trigger day, number of oocytes retrieval, E_2_, P and P/E_2_ on the day before ET in women undergoing IVF/ICSI. Neither free nor total 25(OH)VitD seems to play a major role in human embryo implantation.

## Data availability statement

The original contributions presented in the study are included in the article/**Supplementary Material**. Further inquiries can be directed to the corresponding authors.

## Ethics statement

The studies involving human participants were reviewed and approved by ethics committee of the Reproductive and Genetic Hospital of CITIC-Xiangya (approval number: LL-SC-2018-014). The patients/participants provided their written informed consent to participate in this study.

## Author contributions

Conceptualization: BH; data curation: MT, SZ, SC, CY, WK, KC, YJ, MQT, and YZ; formal analysis: MT and SZ; methodology: MT and SZ; project administration: JL, FG, and BH; software: SZ, CR, and XZ; supervision: GL, FG, and BH; validation: MT, SZ, and BH; writing-original draft: MT and SZ. All authors contributed to the article and approved the submitted version.

## Funding

This study was partially supported by research grants from the Natural Science Foundation of Hunan Province (NO. 2021JJ40372), Huxiang Young Talents project (Grant No. 2021RC3094) and College Student Innovation and Entrepreneurship Training Program of Hunan Province (S202110542051).

## Acknowledgments

BH is the guarantor of this study, he is the person who takes full responsibility for the work as a whole, including the study design, access to data, and the decision to submit and publish the manuscript.

## Conflict of interest

The authors declare that the research was conducted in the absence of any commercial or financial relationships that could be construed as a potential conflict of interest.

## Publisher’s note

All claims expressed in this article are solely those of the authors and do not necessarily represent those of their affiliated organizations, or those of the publisher, the editors and the reviewers. Any product that may be evaluated in this article, or claim that may be made by its manufacturer, is not guaranteed or endorsed by the publisher.
