# Supplementary material for: 25(OH)VitD and human endocrine and functional fertility parameters in women undergoing IVF/ICSI
Source: Front Endocrinol (Lausanne). 2022 Aug 29;13:986848. doi: 10.3389/fendo.2022.986848 (PMC9464865; doi:10.3389/fendo.2022.986848)
Supplement: Supplementary file 1 [file DataSheet_1.docx]

**Supplemental Figures**


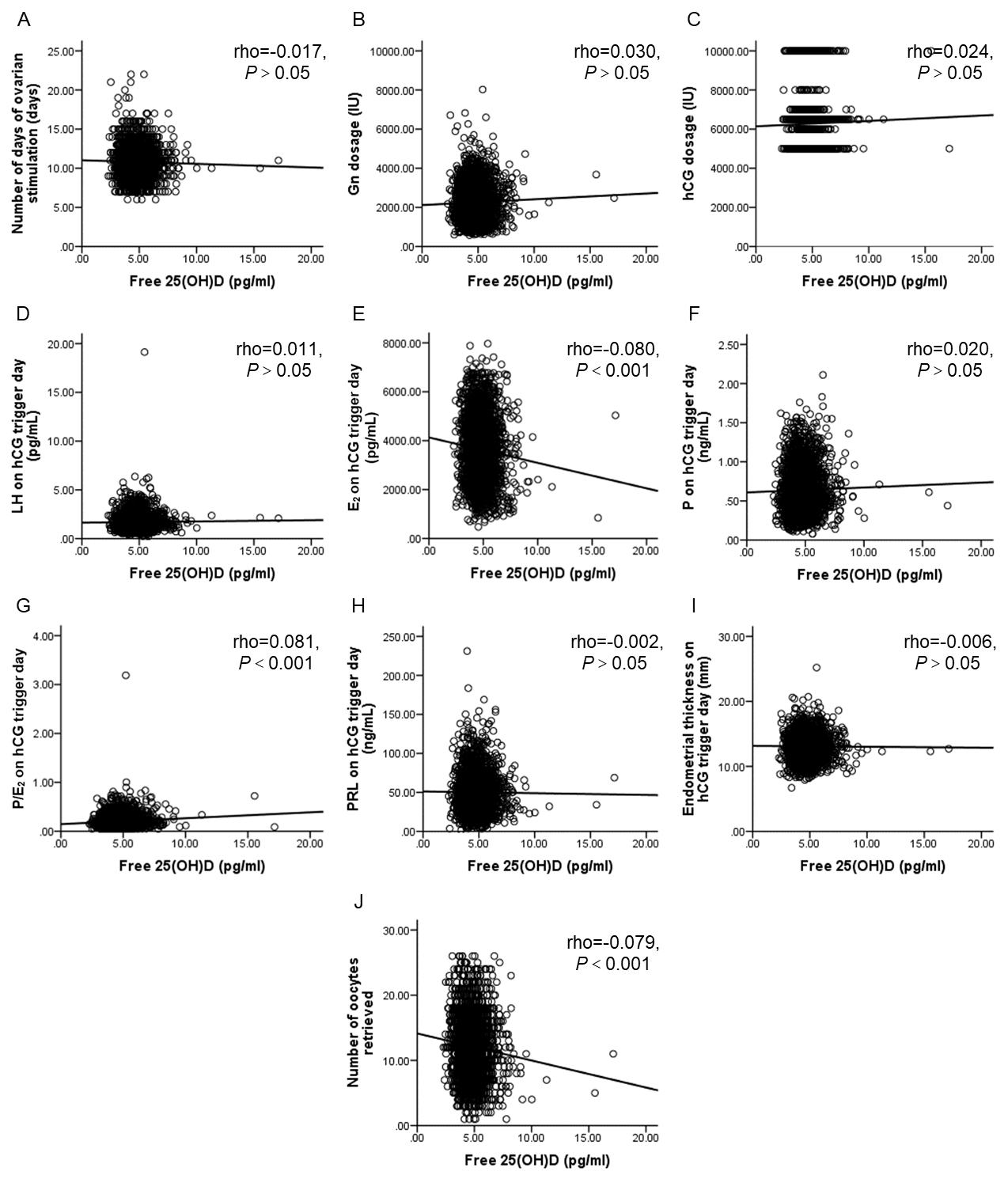


**Supplementary Figure 1 Correlation of free 25(OH)D with COH outcomes**

Abbreviation: 25(OH)D, 25-hydroxyvitamin D; COH, controlled ovarian hyperstimulation; Gn, gonadotropin; hCG, human chorionic gonadotropin; LH, luteinizing hormone; E_2_, estradiol; P, progesterone; PRL, prolactin.

**
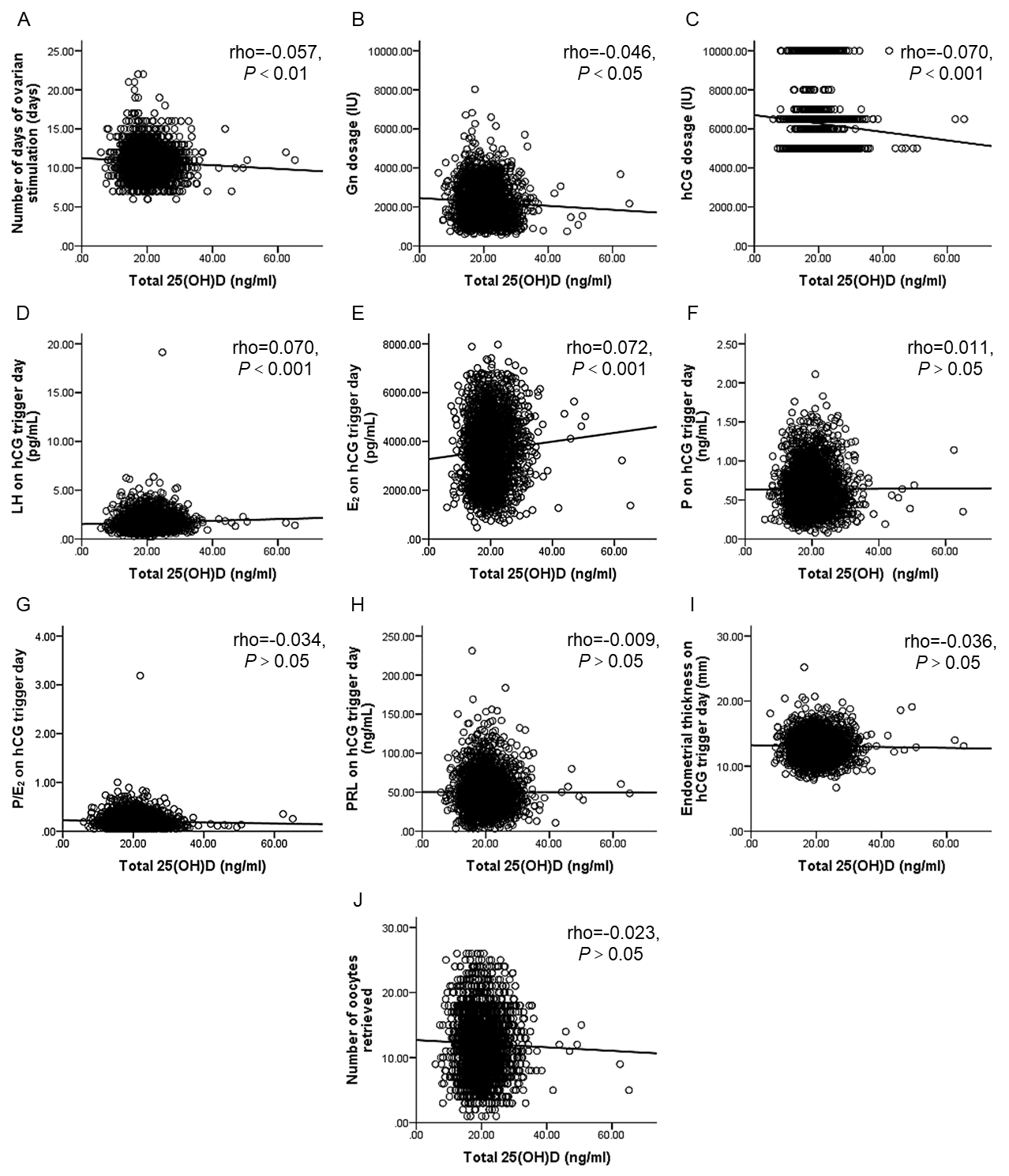
**

**Supplementary Figure 2 Correlation of total 25(OH)D with COH outcomes**

Abbreviation: 25(OH)D, 25-hydroxyvitamin D; COH, controlled ovarian hyperstimulation; Gn, gonadotropin; hCG, human chorionic gonadotropin; LH, luteinizing hormone; E_2_, estradiol; P, progesterone; PRL, prolactin.

**
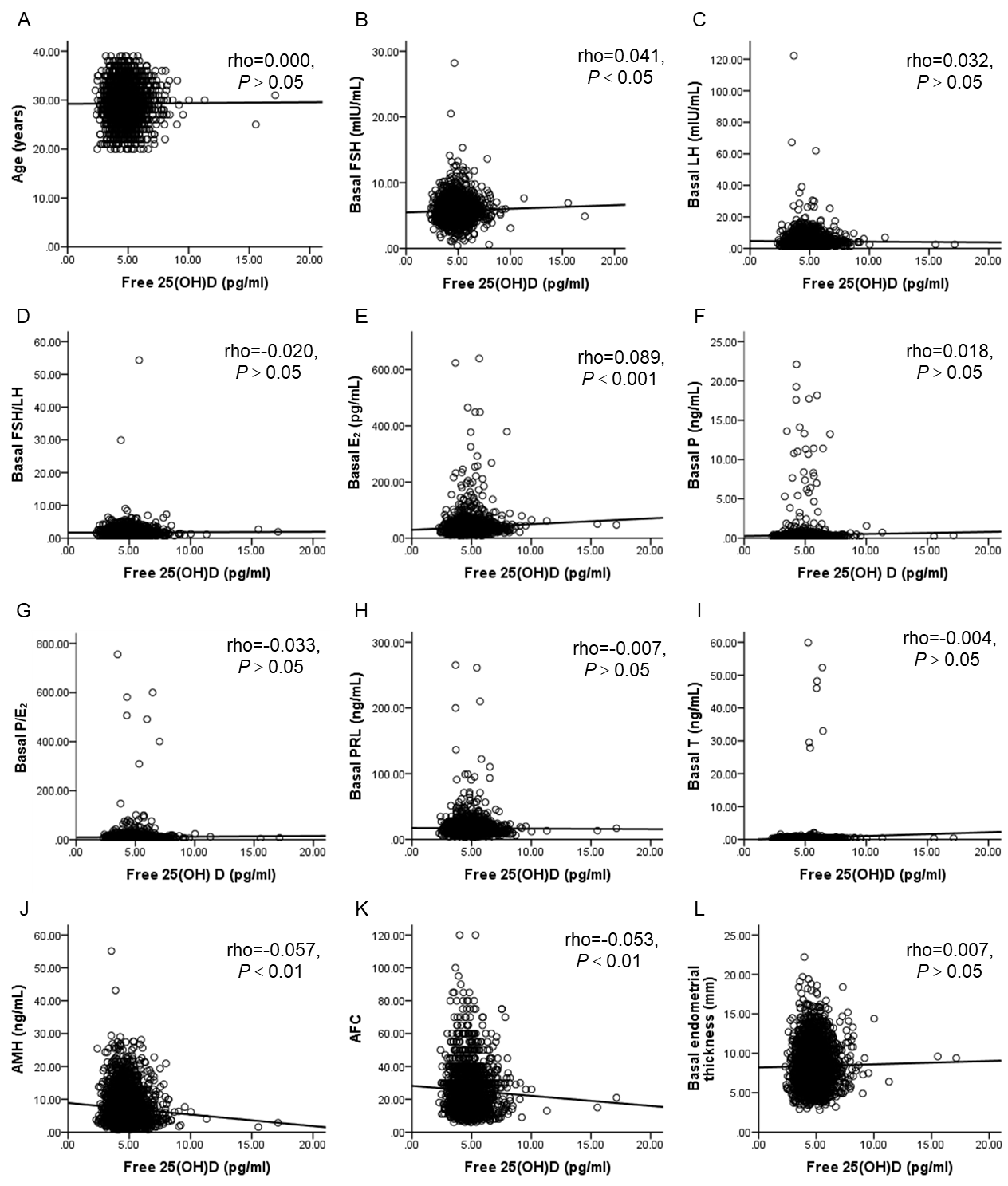
**

**Supplementary Figure 3 Correlation of free 25(OH)D with basal fertility parameters**

Abbreviation: 25(OH)D, 25-hydroxyvitamin D; FSH, follicle-stimulating hormone; LH, luteinizing hormone; E_2_, estradiol; P, progesterone; PRL, prolactin; T, testosterone; AMH, anti-Müllerian hormone; AFC, antral follicle count.


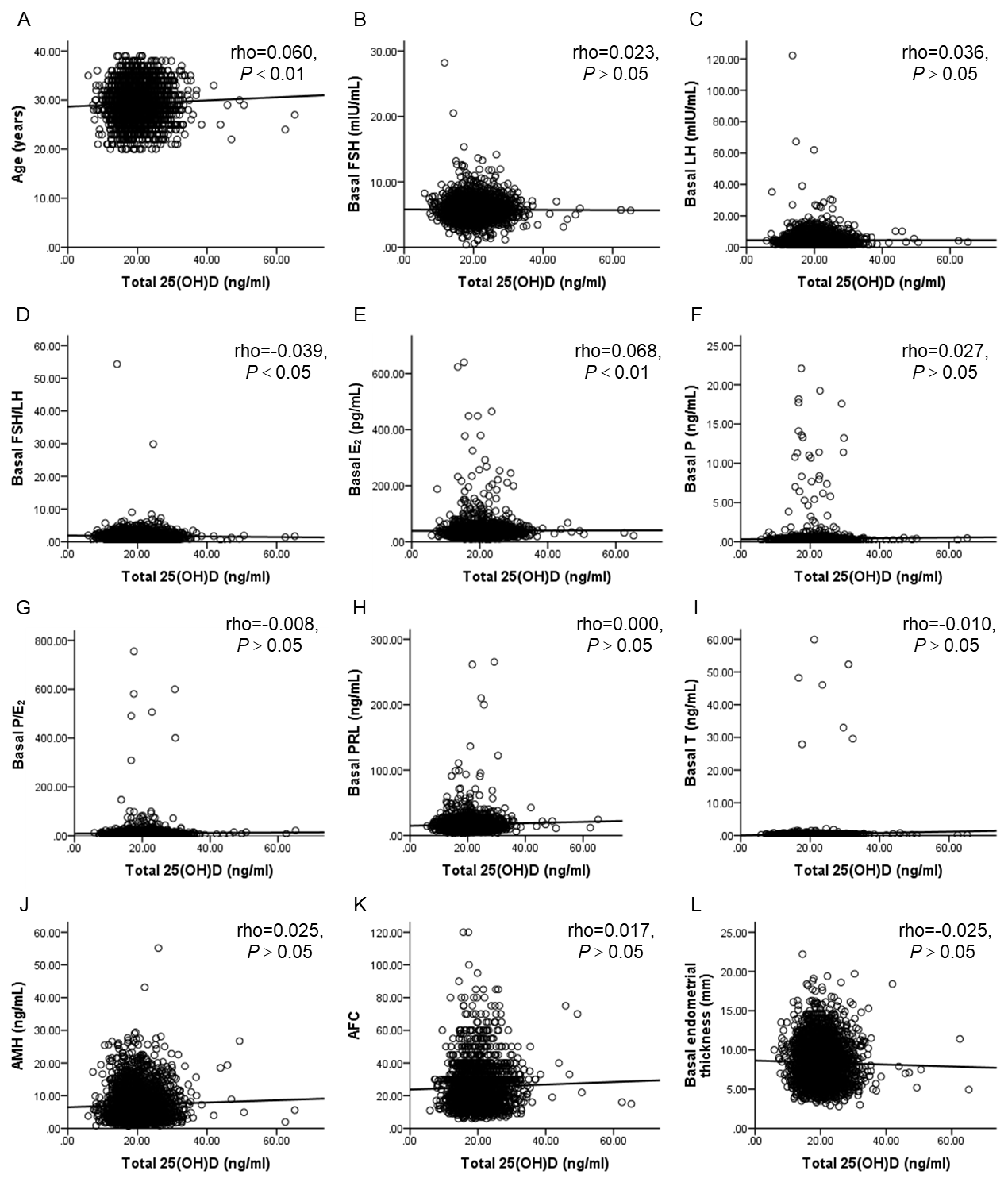


**Supplementary Figure 4 Correlation of total 25(OH)D with basal fertility parameters**

Abbreviation: 25(OH)D, 25-hydroxyvitamin D; FSH, follicle-stimulating hormone; LH, luteinizing hormone; E_2_, estradiol; P, progesterone; PRL, prolactin; T, testosterone; AMH, anti-Müllerian hormone; AFC, antral follicle count.
