# Supplementary material for: 25(OH)VitD and human endocrine and functional fertility parameters in women undergoing IVF/ICSI
Source: Front Endocrinol (Lausanne). 2022 Aug 29;13:986848. doi: 10.3389/fendo.2022.986848 (PMC9464865; doi:10.3389/fendo.2022.986848)
Supplement: Supplementary file 2 [file Table_1.docx]

**Supplementary Table S1**

**Correlation of free 25(OH)VitD and total 25(OH)VitD with baseline fertility parameters and controlled ovarian hyperstimulation outcomes**

|  | **Free 25(OH)VitD (pg/mL)** | | | **Total 25(OH)VitD (ng/mL)** | | |
| --- | --- | --- | --- | --- | --- | --- |
|  | F1 (＜4.32) | F2 (4.32＜~＜5.11) | F3 (≥5.11) | T1 (＜20) | T2 (20＜~＜30) | T3 (≥30) |
| Age (years) | 29.00 (27.00-32.00) | 29.00(27.00-31.00) | 29.00(27.00-32.00) | 29.00 (27.00-31.00) | 29.00 (27.00-32.00) | 29.00 (26.75-32.00) |
| Basal FSH (mIU/mL) | 5.56(4.75-6.44) | 5.60(4.78-6.45) | 5.70(4.82-6.67) | 5.59 (4.76-6.49) | 5.65 (4.84-6.55) | 5.72 (4.88-6.80) |
| Basal LH (mIU/mL) | 3.63(2.59-5.04) | 3.44 (2.57-4.72) | 3.72 (2.69-5.19) ^#^ | 3.55 (2.57-4.85) | 3.66 (2.71-5.10) | 3.42 (2.40-5.42) |
| Basal FSH/LH | 1.55(1.10-2.12) | 1.60(1.14-2.25) | 1.54(1.07-2.08) | 1.60(1.14-2.19) | 1.54(1.06-2.07) | 1.62(1.09-2.24) |
| Basal E_2_ (pg/mL) | 33.00(26.00-41.00) | 33.00(26.00-43.00) | 35.00(29.00-45.00) ^★#^ | 33.00 (26.00-42.00) | 34.00 (28.00-44.00) ^&^ | 34.50 (28.00-43.00) |
| Basal P (ng/mL) | 0.23 (0.17-0.33) | 0.24 (0.17-0.32) | 0.24 (0.18-0.33) | 0.23 (0.17-0.32) | 0.24 (0.18-0.33) | 0.24 (0.16-0.31) |
| Basal P/E_2_ | 7.29 (5.00-10.69) | 7.24 (4.78-10.40) | 7.06 (4.70-10.26) | 7.24 (4.88-10.56) | 7.22 (4.71-10.48) | 6.12 (4.65-9.05) |
| Basal PRL (ng/mL) | 14.99 (11.14-19.82) | 14.81 (10.96-20.22) | 14.51 (10.94-19.84) | 14.91 (11.11-19.81) | 14.52 (10.89-19.86) | 15.87 (12.00-22.49) |
| Basal T (ng/mL) | 0.28(0.22-0.36) | 0.28 (0.23-0.35) | 0.28 (0.22-0.37) | 0.28 (0.23-0.36) | 0.28 (0.22-0.36) | 0.28 (0.22-0.38) |
| AMH (ng/mL) | 5.83 (3.78-9.69) | 5.58(3.64-9.18) | 5.68(3.58-9.08) | 5.68 (3.60-9.09) | 5.69 (3.67-9.50) | 5.98 (3.84-10.08) |
| AFC | 25.00(17.00-30.00) | 23.00 (16.00-30.00) | 23.00 (16.00-30.00) | 23.00 (16.00-30.00) | 24.00 (16.00-30.00) | 27.00 (18.75-33.00) |
| Basal endometrial thickness (mm) | 8.10(6.40-9.80) | 8.10 (6.50-10.00) | 8.10 (6.30-10.13) | 8.20 (6.40-10.00) | 8.00 (6.30-9.90) | 7.55 (5.98-10.43) |
| Number of days of ovarian stimulation (days) | 11.00 (10.00-12.00) | 11.00(10.00-12.00) | 11.00 (10.00-12.00) | 11.00 (10.00-12.00) | 11.00 (10.00-12.00) | 11.00 (9.00-12.00) |
| Gn dosage (IU) | 2100.00 (1537.50-2821.88) | 2100.00 (1537.50-2850.00) | 2175.00 (1537.50-2925.00) | 2175.00 (1575.00-2887.5) | 2100.00 (1500.00-2850.00) | 2081.25 (1453.13-2550.00) |
| hCG dosage (IU) | 6500.00  (5000.00-6500.00) | 6500.00 (5000.00-6500.00) | 6500.00 (5000.00-6500.00) | 6500.00 (5000.00-6500.00) | 6500.00 (5000.00-6500.00) | 5500.00 (5000.00-6500.00) * |
| LH on hCG trigger day (pg/mL) | 1.55 (1.18-1.99) | 1.52 (1.20-1.96) | 1.57 (1.21-2.00) | 1.51 (1.17-1.97) | 1.59 (1.23-2.03) ^&^ | 1.67 (1.31-1.94) |
| E_2_ on hCG trigger day (pg/mL) | 3724.00 (2724.50-4669.75) | 3631.00 (2593.00-4565.00) | 3439.00 (2405.50-4561.25) ^★^ | 3485.00 (2486.00-4493.00) | 3691.50 (2676.75-4696.25) ^&^ | 3896.00 (2793.50-4990.25) * |
| P on hCG trigger day (ng/mL) | 0.59 (0.43-0.80) | 0.59 (0.43-0.79) | 0.58 (0.44-0.80) | 0.59 (0.44-0.78) | 0.59 (0.43-0.81) | 0.56 (0.36-0.76) |
| P/E_2_ on hCG trigger day | 0.17 (0.11-0.24) | 0.17 (0.12-0.25) | 0.18 (0.12-0.27) ^★^ | 0.18 (0.12-0.26) | 0.17 (0.12-0.25) | 0.14 (0.11-0.21) |
| PRL on hCG trigger day (ng/mL) | 47.38 (32.84-63.30) | 47.06 (35.25-60.93) | 46.64 (33.52-63.12) | 47.22 (34.29-62.92) | 46.80 (33.49-61.54) | 48.98(34.75-65.75) |
| Endometrial thickness on hCG trigger day (mm) | 13.10 (11.80-14.40) | 13.00 (11.80-14.30) | 13.00 (12.00-14.30) | 13.20 (11.90-14.50) | 12.90 (11.80-14.20) ^&^ | 12.95 (11.97-14.50) |
| Number of oocytes retrieved | 12.00 (9.00-16.00) | 12.00 (9.00-15.00) | 11.00 (8.00-15.00) ^★^ | 12.00 (9.00-16.00) | 12.00 (9.00-15.00) | 11.00 (8.00-14.25) |

Abbreviation: 25(OH)VitD, 25-hydroxy25(OH)VitD; FSH, follicle-stimulating hormone; LH, luteinizing hormone; E_2_, estradiol; P, progesterone; PRL, prolactin; T, testosterone; AMH, anti-Müllerian hormone; AFC, antral follicle count; COH, controlled ovarian hyperstimulation; Gn, gonadotropin; hCG, human chorionic gonadotropin.

^★^F1 vs F3, *P*＜0.05; ^#^F2 vs F3, *P*＜0.05; ^&^T1 vs T2, *P*<0.05; *T1 vs T3, *P*＜0.05
